# PSF toolkit: an R package for pathway curation and topology-aware analysis

**DOI:** 10.3389/fgene.2023.1264656

**Published:** 2023-08-23

**Authors:** Siras Hakobyan, Ani Stepanyan, Lilit Nersisyan, Hans Binder, Arsen Arakelyan

**Affiliations:** ^1^ Bioinformatics Group, Institute of Molecular Biology, Armenian National Academy of Sciences, Yerevan, Armenia; ^2^ Armenian Bioinformatics Institute (ABI), Yerevan, Armenia; ^3^ Armenian Bioinformatics Institute, Yerevan, Armenia; ^4^ Interdisciplinary Centre for Bioinformatics, University of Leipzig, Leipzig, Germany; ^5^ Russian-Armenian University, Yerevan, Armenia

**Keywords:** biological networks, systems biology, pathway analysis, gene expression, single cell omics

## Abstract

Most high throughput genomic data analysis pipelines currently rely on over-representation or gene set enrichment analysis (ORA/GSEA) approaches for functional analysis. In contrast, topology-based pathway analysis methods, which offer a more biologically informed perspective by incorporating interaction and topology information, have remained underutilized and inaccessible due to various limiting factors. These methods heavily rely on the quality of pathway topologies and often utilize predefined topologies from databases without assessing their correctness. To address these issues and make topology-aware pathway analysis more accessible and flexible, we introduce the PSF (Pathway Signal Flow) toolkit R package. Our toolkit integrates pathway curation and topology-based analysis, providing interactive and command-line tools that facilitate pathway importation, correction, and modification from diverse sources. This enables users to perform topology-based pathway signal flow analysis in both interactive and command-line modes. To showcase the toolkit’s usability, we curated 36 KEGG signaling pathways and conducted several use-case studies, comparing our method with ORA and the topology-based signaling pathway impact analysis (SPIA) method. The results demonstrate that the algorithm can effectively identify ORA enriched pathways while providing more detailed branch-level information. Moreover, in contrast to the SPIA method, it offers the advantage of being cut-off free and less susceptible to the variability caused by selection thresholds. By combining pathway curation and topology-based analysis, the PSF toolkit enhances the quality, flexibility, and accessibility of topology-aware pathway analysis. Researchers can now easily import pathways from various sources, correct and modify them as needed, and perform detailed topology-based pathway signal flow analysis. In summary, our PSF toolkit offers an integrated solution that addresses the limitations of current topology-based pathway analysis methods. By providing interactive and command-line tools for pathway curation and topology-based analysis, we empower researchers to conduct comprehensive pathway analyses across a wide range of applications.

## 1 Introduction

Biological signaling pathways are spatially and temporally distributed series of biomolecular interactions that transduce information in a directional manner and elicit changes in cellular physiology (Azeloglu and Iyengar, 2015). Within a cell, pathways form highly interconnected complex systems, comprising thousands of nodes (proteins, nucleic acids, metabolites, ions) and hundreds of thousands of interactions. This interconnectedness allows for cross-connectivity and cross-talk, ensuring information redundancy, which is crucial for normal cellular function (Lill et al., 2019). Perturbations in signaling pathway activities have been implicated in many complex diseases, including cancers (McCleary-Wheeler et al., 2012; Akbari Dilmaghani et al., 2021; Masliantsev et al., 2021). Thus, the pathway-level analysis of multi-omics data is important for inferring molecular mechanisms underlying disease development and progression in complex diseases and developmental biology (Zhao et al., 2008; Arakelyan et al., 2016; Arakelyan et al., 2017).

Pathway analysis tools can be classified into two main categories. The first category includes overrepresentation and gene set enrichment analysis tools (ORA/GSEA) and their variations, which treat pathways solely as gene sets (or lists). The advantage of these approaches is the ability to handle any pathway or interaction network in a simple and straightforward way. However, they disregard information about pathway component interactions and functional outcomes. The second category comprises tools that leverage pathway topology and protein interaction information which allow for the analysis of information flow within a pathway (Bayerlová et al., 2015; Ansari et al., 2017; Ma et al., 2019). These methods consider both the overall structure of the pathway or interaction network and the specific interactions among its components. By combining this information with the abundance of pathway components measured through high-throughput approaches such as gene expression, proteomics, and metabolomics, comprehensive understanding of the system’s behavior and the molecular mechanisms underlying biological processes can be attained. Several topology-aware tools are currently available, such as SPIA and netGSEA (Tarca et al., 2009; Hellstern et al., 2021). Nonetheless, these tools often utilize pathway topologies “as-is” and are constrained to a set of built-in pathways, collected from different sources. Additionally, these tools commonly report a single pathway state (upregulated or downregulated), disregarding the fact that the same pathway can exhibit a diverse pattern of branch activation, leading to different, and at times opposing, processes (Arakelyan et al., 2016).

High-quality curated pathways serve as a crucial resource for the accurate performance of topology-aware algorithms. Correcting pathway topology ensures that the pathway accurately represents the known interactions and relationships among its components (Arakelyan and Nersisyan, 2013). It also enhances the interpretability and relevance of the pathway in the context of the research question or experimental data, enabling researchers to focus on the specific interactions that are most relevant to their analysis or hypothesis (Volkan Çakır et al., 2017). Pathway analysis methods often rely on the topological information of pathways to assess their functional significance or identify key components or regulators. Incorrect or incomplete interactions can lead to biased or misleading results (Arakelyan and Nersisyan, 2013). Pathways also may exhibit context-specific variations or modifications under different experimental conditions or specific disease states. Correcting pathway topology enables researchers to incorporate such context-specific information into the analysis (Volkan Çakır et al., 2017).

To overcome these limitations of existing topology-aware tools, we developed a pathway curation and activity analysis package for R. With the PSF (pathway signal flow) toolkit, users can easily modify and improve existing pathway topologies, build new pathways from scratch, and analyze them with the branch and node selective pathway signal flow algorithm to study the activity changes along individual pathway branches. The package offers a comprehensive set of curation and editing tools that allow for the seamless integration of pathways from diverse data sources, and facilitate their transformation into a biologically and computationally useable format for topology-aware analysis.

In several use-cases, we demonstrate the utility of our pathway analysis package.

## 2 Materials and methods

### 2.1 General description

The PSF toolkit consists of two interconnected but independent modules for 1) pathway curation (pathway parsing, creation, and editing), and 2) pathway activity (pathway signal flow, PSF) analysis.

**FIGURE 1:**
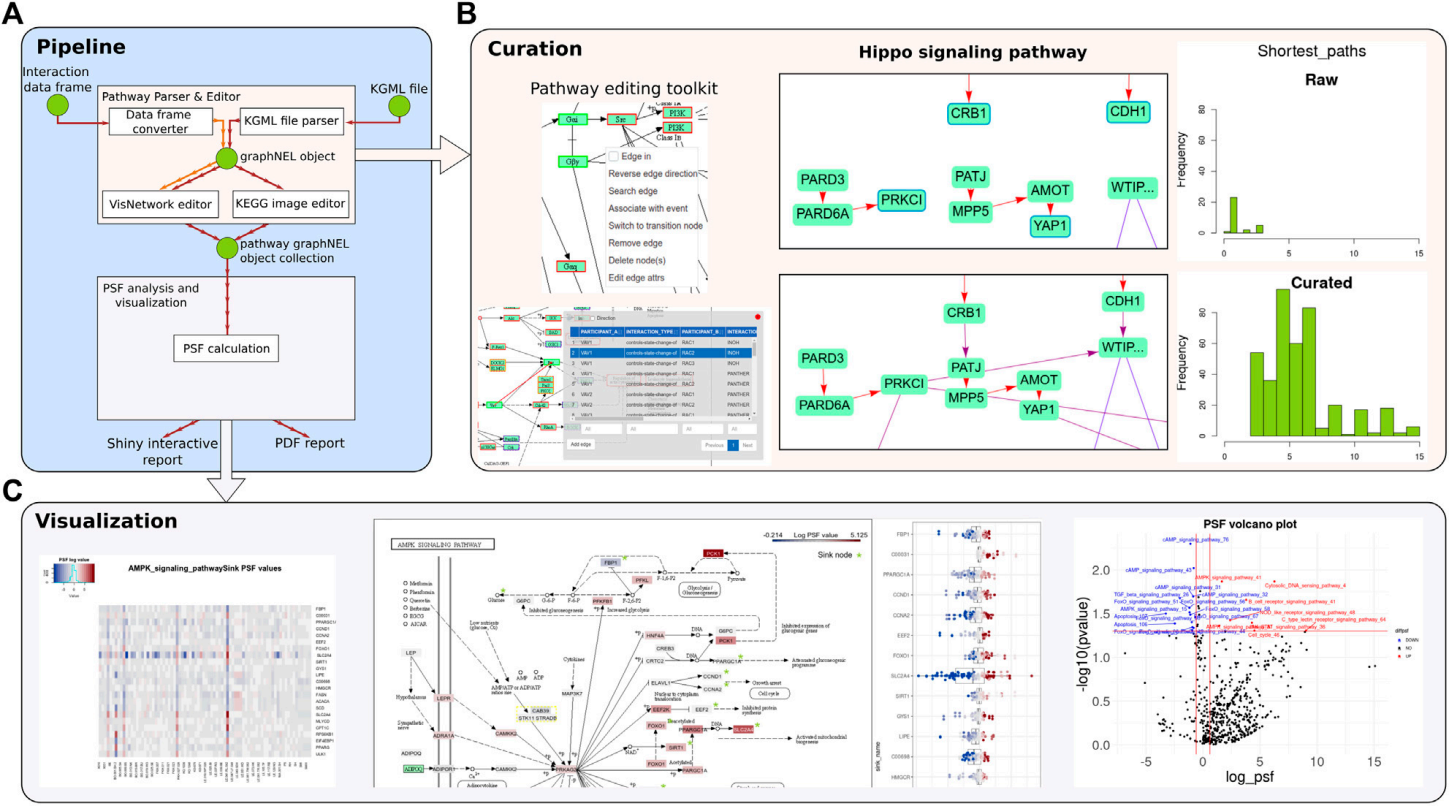
An overview of the PSF toolkit. **(A)** PSF toolkit consists of two major modules for pathway curation and pathway activity analysis/visualization. **(B)** Pathway editing toolkit and a screenshot of the raw and curated Hippo signaling pathway. Barplots represent the number of shortest paths before and after curation. **(C)** The PSF analysis module visualizes pathway activity in terms of a heatmap, topological pathway plots, and a volcano plot for up- and downregulated PSF values.

The toolkit offers both programmatic access in R and a graphical user interface (GUI) as an R Shiny app (see Figure 1A for an overview). With the assistance of the curation module, users can generate analysis-ready pathways with an accurate and context-relevant topology that correctly represent the information flows in the pathway. These pathways can subsequently be used for PSF analysis.

The PSF toolkit package has been tested on three independent platforms (Windows, macOS, Ubuntu) using the GitHub Actions automated build and testing system.

### 2.2 Pathway curation

The PSF toolkit comprises an interactive Shiny application designed for the curation and editing of pathways from diverse sources. Pathways can be imported automatically from the KEGG database via the API or as data frames of nodes and interactions from other databases. Alternatively, users can build pathways from scratch using the integrated “visNetwork” editor.

The Shiny app has two visualization modes: overlay of the pathway graph object on the KEGG pathway image with R “magick” and interactive visualization with the “visNetwork” R packages (the complete list of package dependencies is listed in the GitHub repository of the package https://github.com/hakobyansiras/psf).

The application offers a range of tools for manipulating pathway topologies, including the addition, editing, and deletion of pathway nodes, modification of pathway edges and their attributes, and the creation of one-to-one and one-to-many edges. Users can search, edit, and refine interactions between selected pairs of nodes or between a given node and all other nodes using an integrated protein-protein interaction (PPI) search engine (Cerami et al., 2011; Licata et al., 2020) (Figure 1B). Furthermore, the application incorporates tools for detecting disconnected nodes, node duplications, and loops. Visualization modes are also available to track user modifications and adjust the pathway layout.

Dedicated tools are provided within the application for parsing and editing KEGG pathways. These tools include a parser for KEGG pathways, which automatically corrects “protein-compound-protein” interactions by adding missing interactions, grouping nodes, and establishing binding directions. The parsing tool is inherited from KEGGParser (for Matlab/Octave) and CyKEGGParser (for Cytoscape), which were previously developed by the authors (Arakelyan and Nersisyan, 2013; Nersisyan et al., 2014). Additional information regarding the automated corrections for pathways can be found in the Supplementary Material of the original KEGGParser paper (Arakelyan and Nersisyan, 2013). The overlay of KEGG pathways on images facilitates more informative pathway visualization and aids in the identification and resolution of inconsistencies between the image and KGML using the editor’s tools.

The detailed description of the pathway curation process is presented as one of the use cases in the Results section.

After curation users can save edited pathways as a collection of graph objects (nodes representing pathway entities, edges representing interactions), use them for further pathway analysis in R, or continue analysis in the interactive mode. The toolkit also allows pathway graph modifications programmatically using the “graph” package.

### 2.3 Pathway signal flow analysis

The PSF algorithm computes the activity state of each node (gene or gene groups) in a pathway based on the relative gene expression values and interactions with upstream nodes (Nersisyan et al., 2017). It has been used intensively in many studies of chronic and malignant diseases (Hopp et al., 2015; Arakelyan et al., 2016; Arakelyan et al., 2017).

The algorithm computes the activity values of each node (node signal) through the branches of the pathway, starting from input nodes (ligands/receptors) and progressing toward the end of the pathway branches (terminal/sink nodes). Node signal values are calculated using the following formula:

$$S_{j}={FC}_{j}\sum_{i\in Inc\left(j\right)}p_{i}\omega_{ij}S_{j}^{K_{ij}}$$

where S_j_ represents the node signal, FC_j_ is the fold change value of a node, S_i_ is the signal of the parent/upstream node, w_ij_ is the weight of interaction between the *j* ^th^ and *i* ^th^ node (with a default value of 1), and K_ij_ is the impact (1 for activation, −1 for inhibition). The proportion of the incoming node signal is calculated using the following formula:

$$p_{i}=\frac{S_{i}}{\sum_{k\in Inc\left(j\right)}S_{k}}$$

The significance of PSF value change at branch or pathway levels can be assessed with bootstrapping by reshuffling either sample labels or gene expression values in the pathway.

PSF analysis of the pathway can be performed both in the Shiny app and in the R environment. With a programmatic approach, users can analyze all the pathways in the collection simultaneously, while the GUI allows for real-time visual analysis of a single pathway.

In the Shiny app, the GUI elements enable to select a pathway, upload expression fold change values, run the analysis, and visualize the results with heatmaps, volcano plots, and interactive pathway plots (Figure 1C.). The results are reported in a PDF format and as R objects.

## 3 Results

### 3.1 Curation of KEGG pathways improves their topology

Most topology-aware pathway analysis tools utilize the KEGG Pathway database as their primary source of topological networks (Tarca et al., 2009; Ansari et al., 2017; Hellstern et al., 2021). Several reasons support the selection of KEGG by these tools. Firstly, KEGG is the most comprehensive pathway database, providing detailed topological information, information flow directions, and regulation types (activation/inhibition). Secondly, compared to complex networks with many components, KEGG offers an intuitive separation of pathways and provides clear pathway layouts. Moreover, it provides rich annotation regarding biological processes associated with the overall pathway and its branches.

However, the KEGG Markup Language (KGML) representation of KEGG pathways suffers from several topology-related flaws, including non-connected nodes, missing interactions, and incorrectly annotated interaction types and directions (Arakelyan and Nersisyan, 2013; Nersisyan et al., 2014). For instance, in the Hippo signaling pathway, many branches are disconnected from their final target nodes, rendering it impossible to perform topological analyses without proper curation (Supplementary Figure S1B). Despite these issues, topology-aware tools typically utilize KGML representations without correction, which can lead to significantly misleading results in topology-aware analysis, especially for many KEGG pathways. There are numerous editors and parsing tools available for KEGG pathways in environments like Cytoscape and R. Some of these tools are outdated and no longer maintained (Klukas and Schreiber, 2007; Wrzodek et al., 2011). Others, such as KEGGscape in Cytoscape and KEGGgraph in R provide pathway parsing and visualization but lack editing and curation functionalities (Zhang and Wiemann, 2009; Nishida et al., 2014). Similarly, although the graphite R package enables pathway import from different databases for topology-aware analysis, it lacks editing, and curation capabilities, and has limited functionality for visualization (Sales et al., 2012). In contrast, the PSF toolkit stands out by encompassing all essential functionalities, including pathway import, interactive editing, curation, and topology-aware analysis.

Using the curation module of our package, we improved 36 signaling pathways from the KEGG database and conducted several use-case studies to demonstrate the applicability of our approach. The manual curation process in the pathway editor was performed with two steps. It has been observed that KGML files often contain inconsistencies compared to pathway images, which serve as the “ground truth” in the KEGG database. In the first step, networks were recovered and curated based on pathway image information. Image-based visualization enables quick detection and resolution of inconsistencies between the image and KGML using the editor’s tools. Fixes made during this step include recovering missing interactions in KGML files based on pathway images, correcting wrong interaction directions, removing extra parts of pathways that are outside the main network, and adding nodes for processes and events present only in the pathway images. The second step of manual curation involved addressing more complex cases that cannot be resolved solely using the pathway image. The second part of curation was performed with the help of a literature review and the use of an integrated PPI database.

**FIGURE 2:**
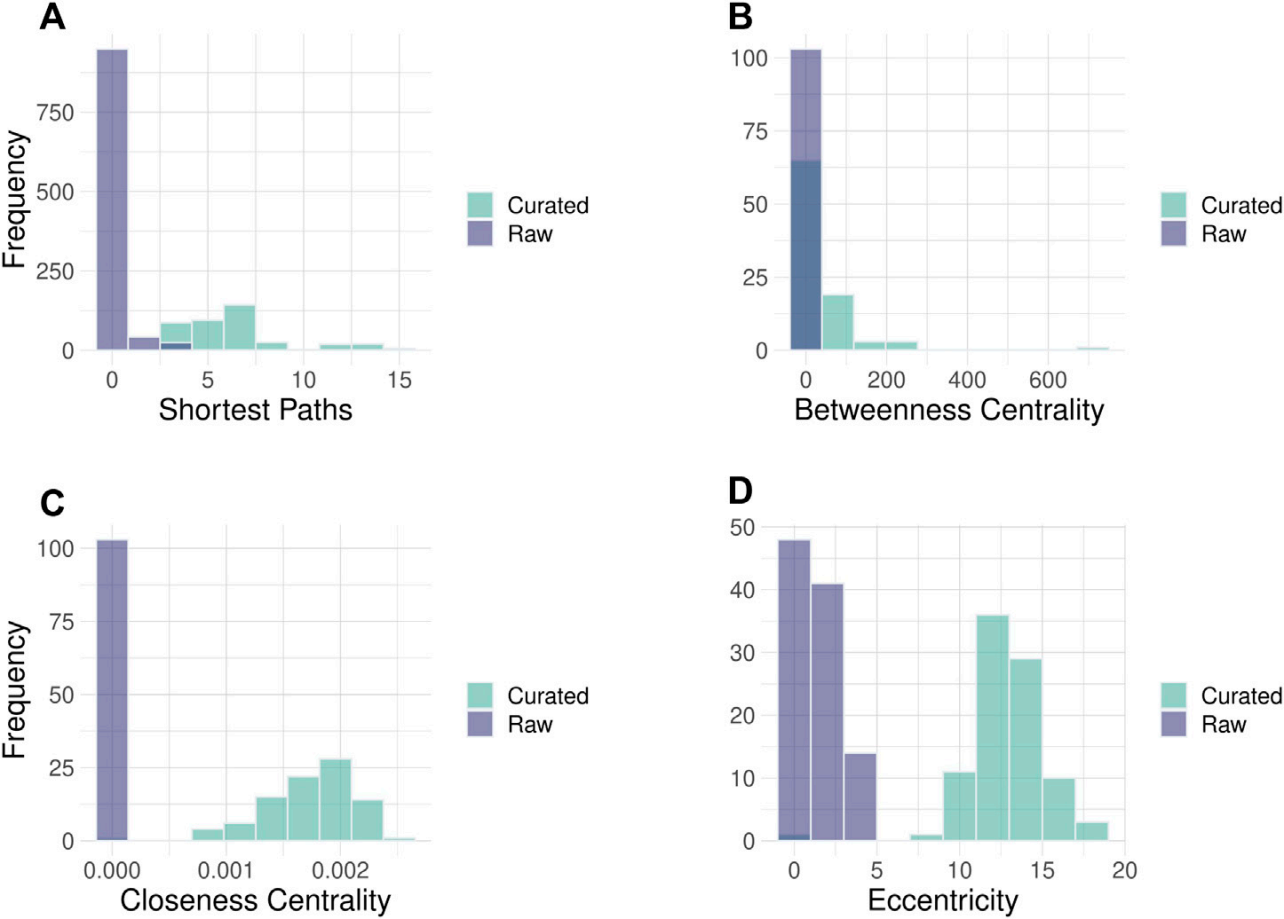
Histograms of node parameters for Hippo signaling pathway in raw and curated states. Improvement of the network topology parameters can be noted in curated pathways. **(A)** Number of shortest paths between pathway nodes. The shortest path is the minimal number of edges that need to be traversed to reach from source to the target node. **(B)** The closeness centrality of nodes measures their average farness from all other nodes. **(C)** The Betweenness centrality measures how often a node occurs on all shortest paths between two nodes. **(D)** The eccentricity centrality shows how easily accessible a node is from any other node in the network. The images of raw and curated pathways are available in the Supplement.

We demonstrate here the improvements in Hippo signaling pathway topology. After automatic error correction during parsing (18 fixes), additional curations (24 fixes, including fixing the binding edge direction, and restoring missing edges) were performed interactively in the toolkit’s GUI (Figure 2; Supplementary Figure S1). Overall, 188 interaction directions, 553 missing interactions, and 168 group nodes were fixed in the 36 pathways. This considerably improved the topological properties of the pathways particularly the overall connectivity and increased diameter of the pathways (Table 1). The curated pathways are available in the R package and can be used for downstream PSF analysis.

**TABLE 1:** The median values of network topology parameters in original and curated KEGG pathways^a^.

	Original pathways (N = 36)	Curated pathways (N = 36)
Shortest path (the minimal number of edges that need to be traversed to reach from source to the target node.)	84	180
Betweenness (the frequency of a node occurrence on all shortest paths between two nodes)	0	2.04
Eccentricity centrality (accessibility of a node from any other node in the network)	6	9
Diameter (the length of the shortest path between the most distanced nodes)	7	11
Disconnected nodes (the number of nodes without incoming and outgoing edges)	10.5	0
Sink (terminal) nodes (Nodes without outgoing edges)	20	14.5

^a^ The table presents network properties which describe connectivity, overall completeness, and quality of pathways. A detailed description of biological network parameters is provided in (Koutrouli et al., 2020).

### 3.2 Comparing PSF with SPIA

We compared the performance of PSF and one of the most popular topology-based pathway analysis methods - SPIA (Tarca et al., 2009). SPIA is a topology-aware pathway analysis method that combines evidence from overrepresentation analysis and topological analysis to estimate the state of the whole pathway (activated/inhibited). It takes as input a list of significantly differentially expressed genes and their fold change values.

We conducted a pathway analysis to identify differences between primary melanoma and benign melanocytic nevi, using RNA-seq data (GEO accession number GSE112509) (Kunz et al., 2018). Both algorithms were tested on the same set of 36 KEGG signaling pathways with the same gene expression values calculated with the DESeq2 R package (*p* adjusted < 0.05, for SPIA and cutoff-free fold change values for PSF). To calculate PSF significance we used bootstrapping (*n* = 2000) to check the probability of getting the same or more extreme PSF values by chance. The output of SPIA was a ranked list of pathways and their states based on the combined significance of ORA and topology analysis. The output of PSF included the activity of all pathway branches with calculated significance. To compare the results of both methods, we considered the list of significant pathways from SPIA and the list of pathways that had at least one significantly deregulated branch according to PSF.

Four pathways were labeled as differentially deregulated both by SPIA and PSF algorithms. Another four pathways were additionally detected only by SPIA, and 16 pathways were detected only by the PSF algorithm. Examination of PSF-detected significant pathways showed that the majority of deregulated pathways had few or only one deregulated branch. One such example is the AMPK signaling pathway where the “Growth arrest” branch showed a 10-fold activity drop due to the activation of the AMPK gene through the Leptin Receptor (Supplementary Figure S2).

Evaluation of branch-level activity has specific advantages over “total” pathway activity estimated by topology-aware tools such as SPIA, PADOG, and ROntoools (Tarca et al., 2009; 2012; Ansari et al., 2017) because depending on downstream signaling different branches of the same pathway can trigger different processes, sometimes even with opposite effects (see, e.g., Ras signaling pathway, Hippo signaling pathway, Jak-Stat signaling pathway). Thus, one score per pathway might not be sensitive enough to detect, e.g., a dysregulated branch. For instance, the Chemokine signaling pathway was reported with SPIA as activated with 0.05*10-4 *p*-value. A detailed view however shows that, while a large portion of the pathway is indeed activated, three terminal nodes, in contrast, were significantly downregulated because of the inhibitory effect from the activated AKT3 gene (Supplementary Figure S3).

Upon examining the four pathways that were detected only by the SPIA method, it was found that the enrichment of the pathways had the greatest impact, while the topology component was virtually irrelevant. The differentially expressed genes in those pathways meeting significance criterion (*p* <0.05) had only marginal fold change not impacting the overall activity of pathways. After filtering out DEGs with low fold change (logFC>|1|) two out of the four deregulated pathways were removed from the SPIA list. Hence, SPIA is sensitive to threshold settings with consequences for the robustness of the results (Ansari et al., 2017).

### 3.3 Analysis of pathway activity fluctuations on gene and coding isoform levels in melanoma

We used the PSF toolkit to analyze the profiles of signaling pathway activities in melanoma and melanocytic nevi samples at the gene and protein-coding isoform levels. Our objective was to identify pathway-level effects arising from alterations in the content of coding and non-coding isoforms in melanoma. Our previous studies indicated significant differences in gene- and coding isoform-level expression in molecular subtypes of benign melanocytic nevi and primary melanomas (Hakobyan et al., 2021). Since the overall gene expression may not necessarily represent the fraction of isoforms that can be translated into functional proteins, we compared the PSF scores for signaling pathways with total gene expression values and coding isoform expression values only.

Here, we used RNA-seq data of benign melanocytic nevi and primary melanomas deposited in the Gene Expression Omnibus (accession number GSE112509). The RNA-seq raw FASTQ files were processed with Kallisto (Bray et al., 2016), which performs isoform-level quantification of gene expression using Genecode transcript annotation (Release 42) as a reference. Quantified isoform level data were normalized and further used to calculate gene isoform fractions as the ratio between the isoform expression and corresponding gene expression. The total fractions of all isoforms from one gene must sum up to 1.

CPC2 tool was used to detect non-coding isoforms from their sequences (Kang et al., 2017).

**FIGURE 3:**
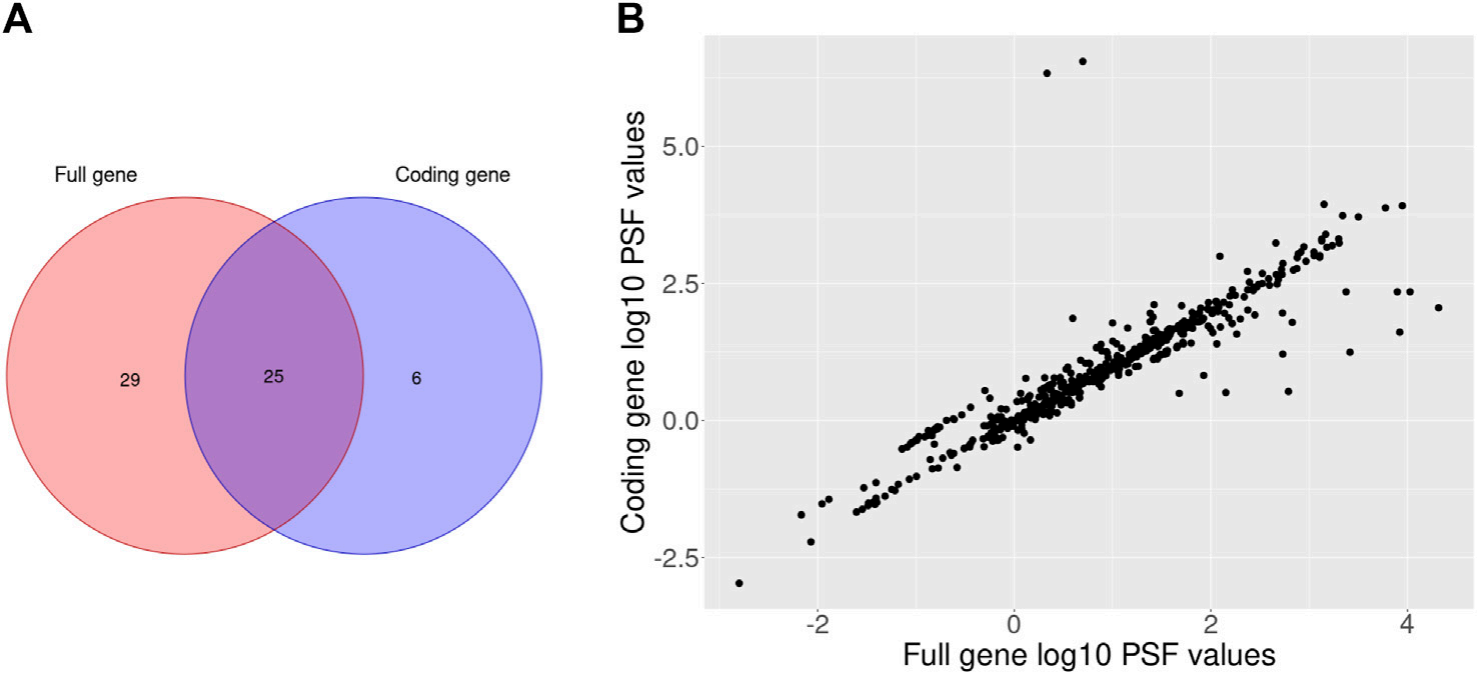
Venn diagram of the significantly deregulated sinks between melanoma type 1 and nevi type 1 on full and coding gene expression levels **(A)** and a scatterplot of full and coding gene expression PSF values for the comparison between melanoma type 1 and nevi type 1 **(B)**.

To filter out the non-coding isoform expression we multiplied the total fraction of coding isoforms with corresponding gene expression values. Next, we performed differential expression analysis between melanoma type 1 and nevi type 1 groups with the DESeq2 R package for full and coding gene expression data. Then, the calculated fold change values were used to perform PSF analysis on 36 curated KEGG signaling pathways. The significance of PSF values was calculated with bootstrap (2000 steps) to check the probability of getting the same or more extreme PSF values by chance. Finally, we compared lists of significantly deregulated pathway sinks between gene and coding isoform expression level results (Figure 3A).

The comparison of full and coding gene expression PSF values revealed a general correlation between pathway activities with few obvious outliers (Figure 3B). The pathways which were significantly deregulated only on coding gene expression level had isoform content change in their genes. Particularly, in the cAMP signaling pathway 9 genes (GRIA1, FXYD1, AKT3, NFATC1, NPY1R, PPP1CB, CAMK4, CFTR, PIK3CA) had switched isoforms (e.g., coding isoforms were replaced with noncoding isoforms while the gene expression remained unchanged in groups) which were detected with IsoformSwitchAnalyzeR R package in our previous study (Hakobyan et al., 2021). Another two pathways (AMPK and JAK-STAT signaling) which were also significantly deregulated on coding gene expression analysis also contained isoform switches in 7 genes (TBC1D1, AKT3, RAB2A, CAB39L, PPP2R5E, CFTR, PIK3CA; AKT3, SOCS6, CCND3, IL3RA, IL3RA, PIK3CA, GHR). Thus, adjustment of gene expression data based on coding isoform expression can affect pathway activity in terms of PSF values.

### 3.4 Analysis of pathway activity using single-cell transcriptome data

Here we were interested in whether our PSF algorithm has utility in single-cell data analysis. The single-cell level transcriptomic analyses can provide, for example, important insights into tumor heterogeneity (Wu et al., 2021), microenvironment (Nieto et al., 2021) as well as organismal development (Shapiro et al., 2013). Here we used the scRNA-seq dataset from the GTEx database (Eraslan et al., 2022) to evaluate the activity of 36 curated signaling pathways and check cell cluster separation on the pathway level. To overcome sparsity issues in PSF analysis we used the pseudo-bulk approach (Crowell et al., 2020) and aggregated expression values from the same tissue types․ From each tissue type 5,000 cells were taken and summed up for every 50 cells. Then, gene expression fold changes were computed against the global mean of pseudo-bulk expressions. Pseudo-bulk transformed expression fold change values were used to analyze the activity of curated signaling pathways with PSF. A total of 614 activity values were calculated for 36 signaling pathways.

We used the Seurat R package (Hao et al., 2021) to compare the sample clustering based on expression and PSF activity values. Both single-cell expression and PSF activity data were log transformed and mean-normalized before clustering.

**FIGURE 4:**
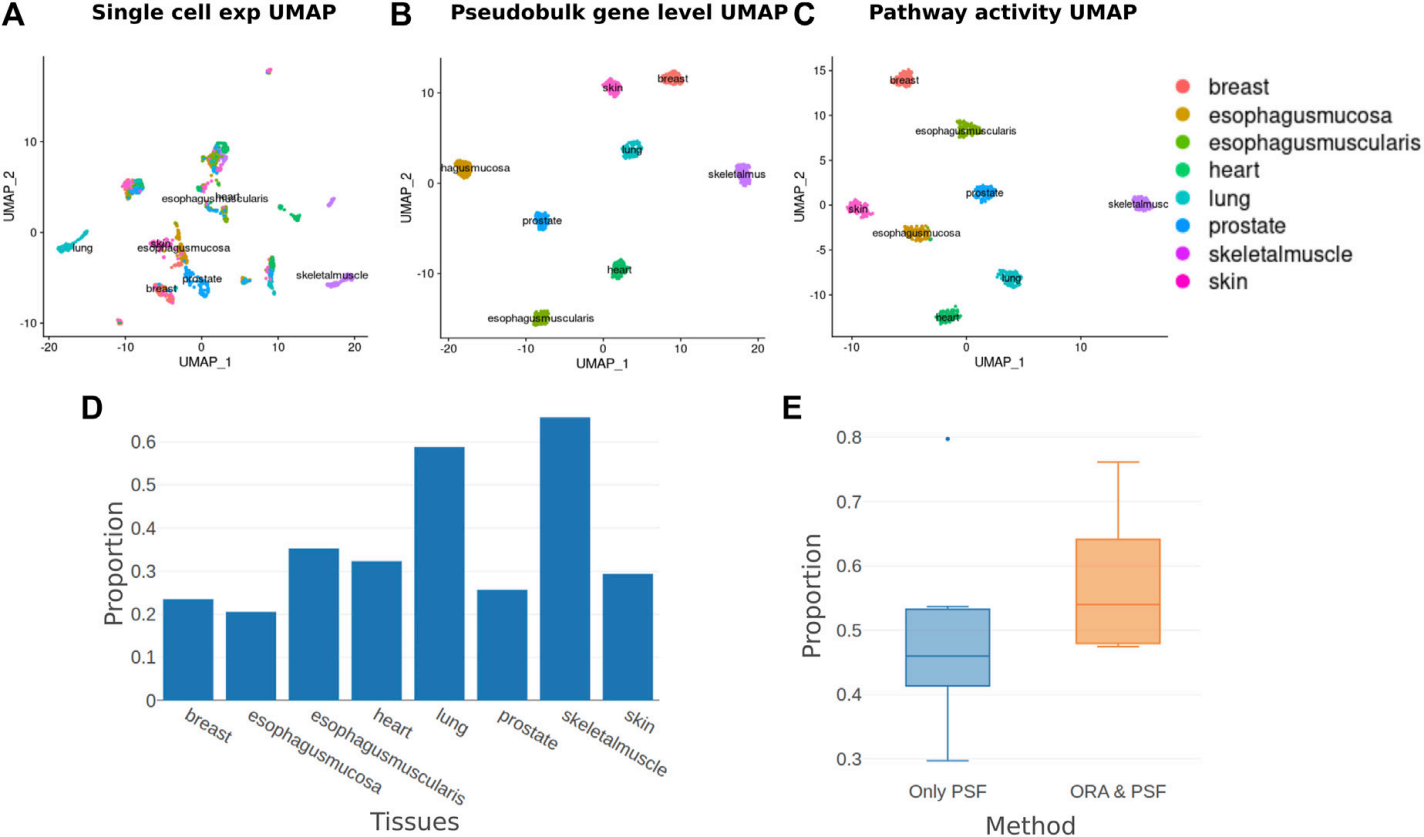
Single-cell pathway analysis clustering and enrichment. **(A)** UMAP plot of normalized single-cell gene expression values. **(B)** UMAP plot of pseudo-bulk transformed single-cell expression values. **(C)** UMAP plot of pathway activities calculated with the PSF algorithm from pseudo-bulk transformed aggregated cell expression values. **(D)** The proportion of pathways detected by ORA from those detected by the PSF method for each tissue. **(E)** Proportions of Deregulated Branches in only PSF detected pathways and pathways detected both with ORA and PSF.

Both gene-level and PSF-level clustering showed a clear separation of tissues (Figures 4A–C). Despite having very similar clustering results, the clusters in the PSF-level analysis explicitly associate with the underlying pathways (Supplementary Table S1). The functional features associated with the clusters were linked to the physiology and function of the corresponding tissue types. Thus, the highest activation of the FoxO signaling pathway was observed in muscle tissues in agreement with its essential role in muscle development and function (Xu et al., 2016). On the other hand, heart cells showed remarkably upregulated activity of the calcium signaling pathway compared with other tissues, where it is known to control important cardiac functions (Terrar, 2020).

To compare gene-level and pathway-level results, we performed an overrepresentation analysis for 36 curated signaling pathways to identify enriched pathways for cluster-specific genes. Then, we compared the list of overrepresented pathways on the gene level with the deregulated pathways detected by PSF for each cluster (Figure 4D). We noticed that ORA detected fewer pathways than PSF in many clusters. Results showed that enriched pathways with the ORA method had on average 57% deregulated branches according to PSF analysis, compared to 48% for non-significant pathways (Figure 4E, see example pathways in Supplementary Figure S4). Thus, the advantage of PSF in this case can be its ability to detect branch level changes.

We demonstrated that utilizing PSF analysis in conjunction with a pseudo-bulking approach effectively reduces the number of analyzed features by leveraging pathway analysis. This reduction in features ensures consistent clustering while offering valuable pathway-branch level information on cluster-specific features.

## 4 Discussion

In this paper, we present the “PSF toolkit,” a novel R tool for topology-aware pathway analysis that integrates pathway curation and analysis modules. The curation platform offers a comprehensive set of tools to curate and enhance pathways from any source. Platform also provides specific tools to enhance existing KEGG pathways by aligning them with image templates and accurately representing signal flows from upstream to downstream nodes. Users can also create pathways from scratch using the interactive Visnetwork editor or import them through node and interaction data frames. In addition to incorporating features from previous curation and pathway analysis packages, the PSF toolkit includes improved functions for report generation, pathway graph manipulation, and enhanced performance compared to Matlab- and Cytoscape-based packages (Arakelyan and Nersisyan, 2013; Nersisyan et al., 2014). Another advantage is its availability in R, which is currently one of the most popular environments for “omics” data analysis.

Pathway analysis has become a widely used method for the functional analysis of high-throughput genomic data (Das et al., 2020; Maleki et al., 2020). In general, we can classify analysis methods into two main categories: enrichment-based methods that utilize gene sets for analysis and topology-based methods that require more detailed information about biological processes, such as interactions and their types. Each of these classes has its own advantages and disadvantages.

The first class of analysis methods, known as enrichment methods, offers several advantages. Firstly, they leverage a vast number of gene sets that potentially cover almost all human genes. Secondly, they offer flexibility in terms of combining genes in various gene sets depending on the biological context. Lastly, these methods employ computationally efficient approaches, making them easily applicable to large datasets. However, enrichment methods neglect the network properties of biological processes as they intentionally disregard the relationships between genes within the gene sets.

The second class of methods explicitly takes into account the graph-structure of biological pathways and considers interactions and their types. These methods enable analysis of signal flow along the pathway from upstream receptors to downstream effectors. However, there are several limitations that hinder the widespread use of these methods. The value of pathway analyses critically depends on the correctness of the pathway-graph. Hence, the primary limitation is the reliance on high-quality, manually curated pathways. Currently, available curated pathway databases with rich topology coverage only encompass approximately 10,000 human genes, which is less than half of the complete set. Additionally, these methods can involve complex analysis approaches that are time-consuming and computationally expensive. Lastly, interpreting the results and visualizing the detailed information can pose challenges with these approaches.

To address some of the limitations associated with topology-aware analysis methods and advance this direction, we have developed the PSF toolkit. The toolkit includes an interactive curation module that tackles data availability issues by allowing quick improvement and curation of pathways from any source. Additionally, the visualization module enables result visualization and facilitates the exploration of disrupted pathways in an interactive manner.

Since the development of the PSF algorithm, it has been applied in several studies to assess pathway activity in malignant and chronic lung diseases (Arakelyan et al., 2016), autoinflammatory and autoimmune disorders (Arakelyan et al., 2017), molecular mechanisms of desiccation tolerance in plants (Mladenov et al., 2022), telomere length maintenance in colorectal cancers (Nersisyan et al., 2019), among others. In this study, we expand the application of the PSF toolkit to the analysis of isoform expression-based and single-cell pathway activity estimation.

Furthermore, we compared the performance of the PSF algorithm with the widely used and cited SPIA algorithm (Tarca et al., 2009). The findings reveal that SPIA tends to identify pathways as significant when they contain a large number of differentially expressed genes (DEGs), irrespective of their topology and interactions. Hence, SPIA is highly sensitive to the DEG selection cut-off and may yield inconsistent results in the case of improper selection. In contrast, our PSF approach offers two advantages over SPIA: cut-off-free analysis and significance calculation at the pathway branch level.

In conclusion, our presented R package, the ‘PSF toolkit,’ offers a comprehensive solution with its pathway signal flow algorithm and interactive curation and visualization platform. The package caters to diverse user preferences by providing both programmatic and GUI access options. Its versatility extends to various applications, including bulk and single-cell expression data analysis, and other gene-level -omics data, ultimately broadening the scope of topology-aware pathway analysis.

## Data Availability

Publicly available datasets were analyzed in this study. This data can be found here: https://gtexportal.org/home/datasets https://www.ncbi.nlm.nih.gov/geo/query/acc.cgi?acc=GSE112509. PSF toolkit is implemented in R and licensed under the GNU General Public Licence v3.0. Source code and documentation are available at: https://github.com/hakobyansiras/psf. All the plots and results of the paper can be reproduced with R scripts provided at this link: https://github.com/hakobyansiras/psf/tree/main/inst/extdata/psf_paper_scripts.
