# Supplementary material for: PSF toolkit: an R package for pathway curation and topology-aware analysis
Source: Front Genet. 2023 Aug 23;14:1264656. doi: 10.3389/fgene.2023.1264656 (PMC10482229; doi:10.3389/fgene.2023.1264656)
Supplement: Supplementary file 1 [file DataSheet1.PDF]

Supplementary Material

A.

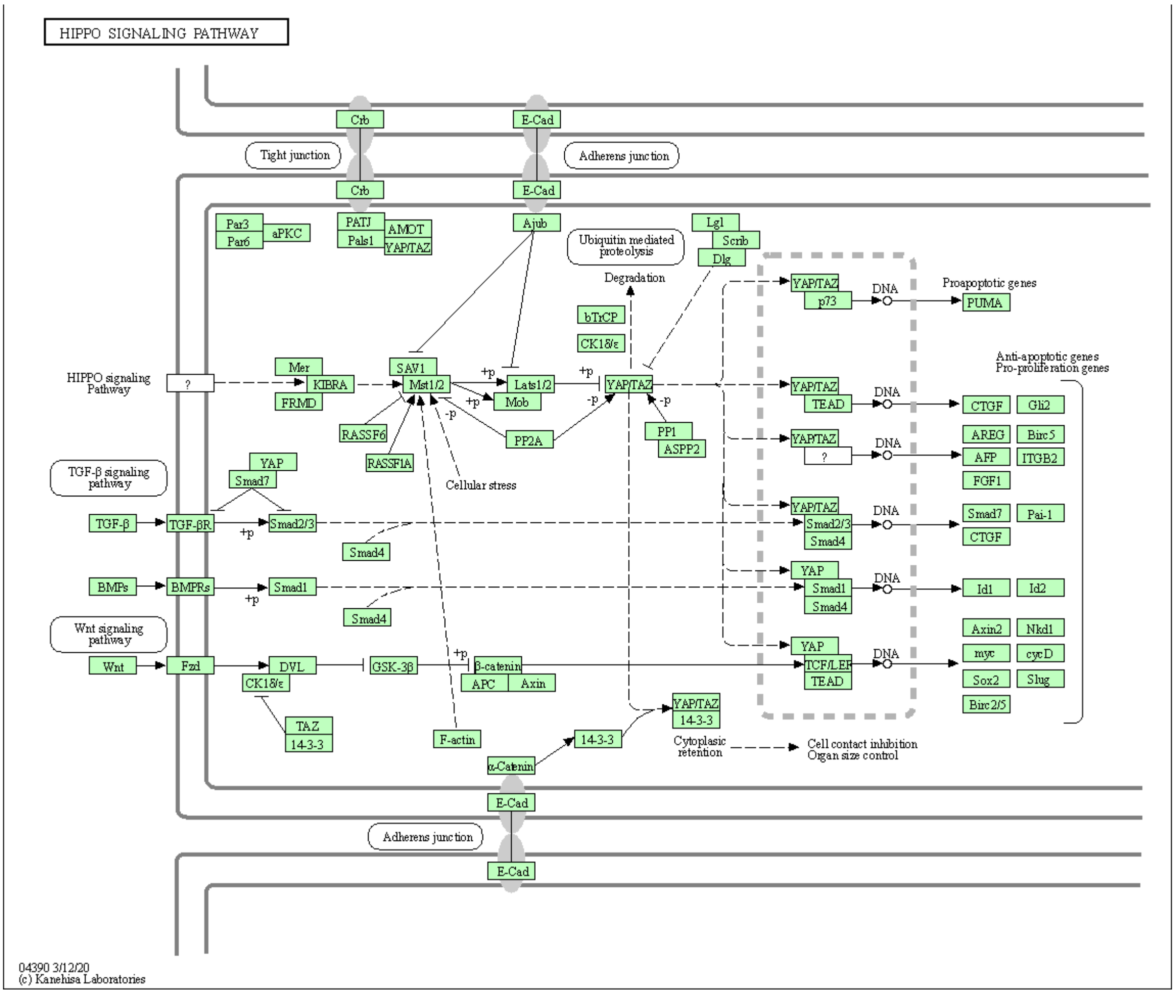

B.

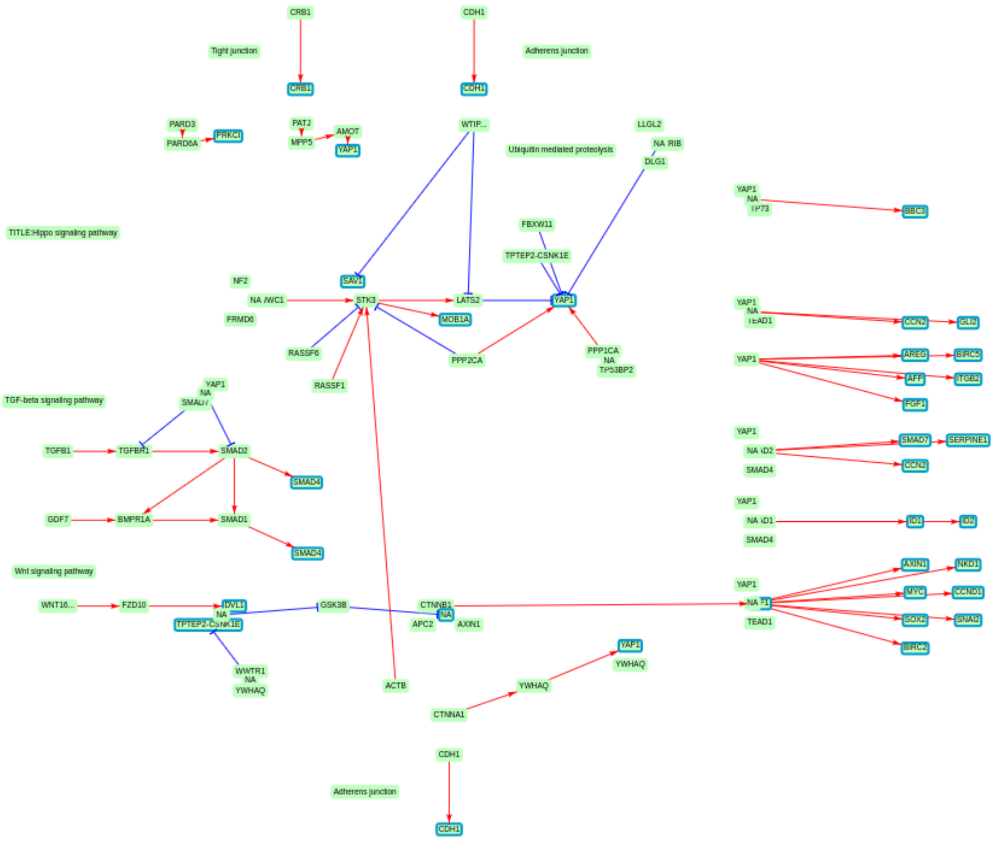

C.

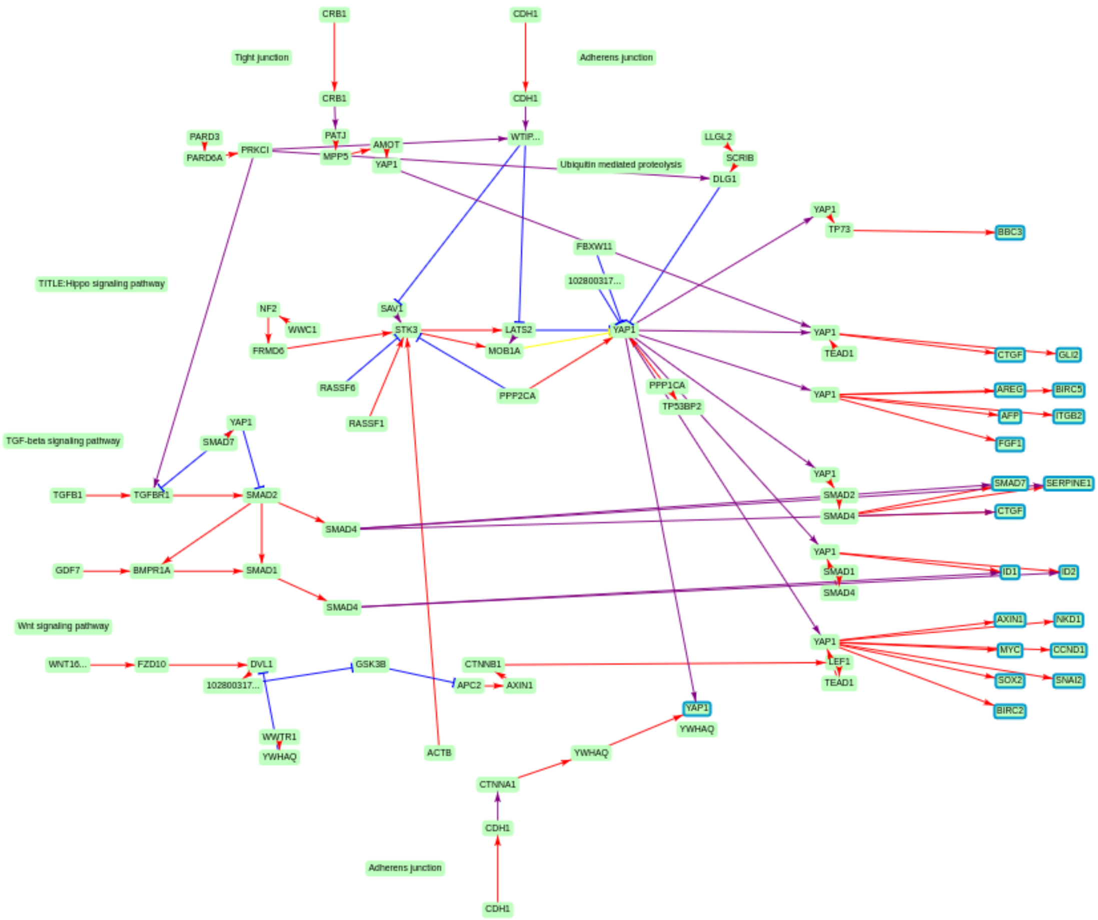

Figure S1. A. Original KEGG pathway image of Hippo signaling pathway. B. Network extracted from the original KGML file. C. Network after automated and further manual curation with the PSF toolkit (18 fixes with automatic error correction during parsing, 24 fixes with manual curation). Nodes with a blue border are terminal (sink) nodes. Red and blue lines between the nodes are activation and inhibition interactions, respectively. Purple and yellow lines are manually curated interactions.

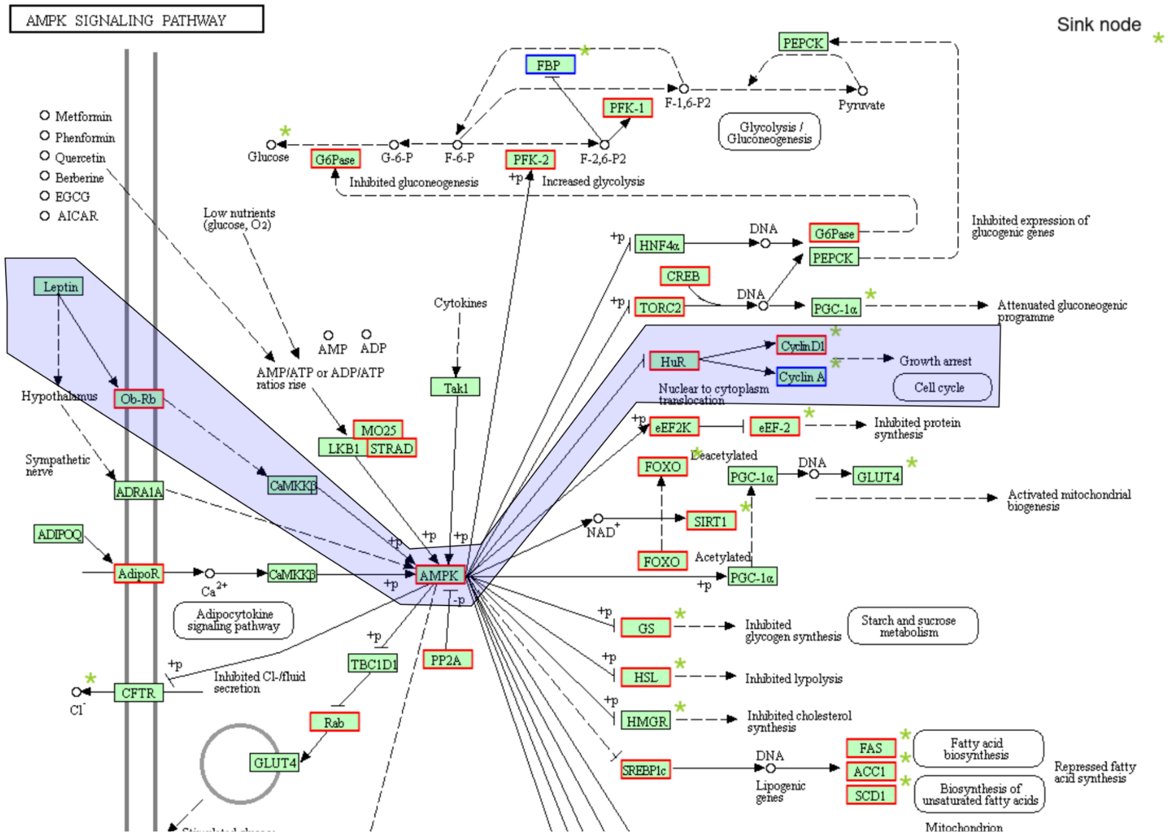

Figure S2. AMPK signaling pathway detected with only PSF. Differentially expressed genes/nodes are highlighted with red borders. According to the PSF algorithm, the blue highlighted branch was downregulated 10-fold due to the activation of the AMPK gene through the Leptin Receptor which represses growth arrest.

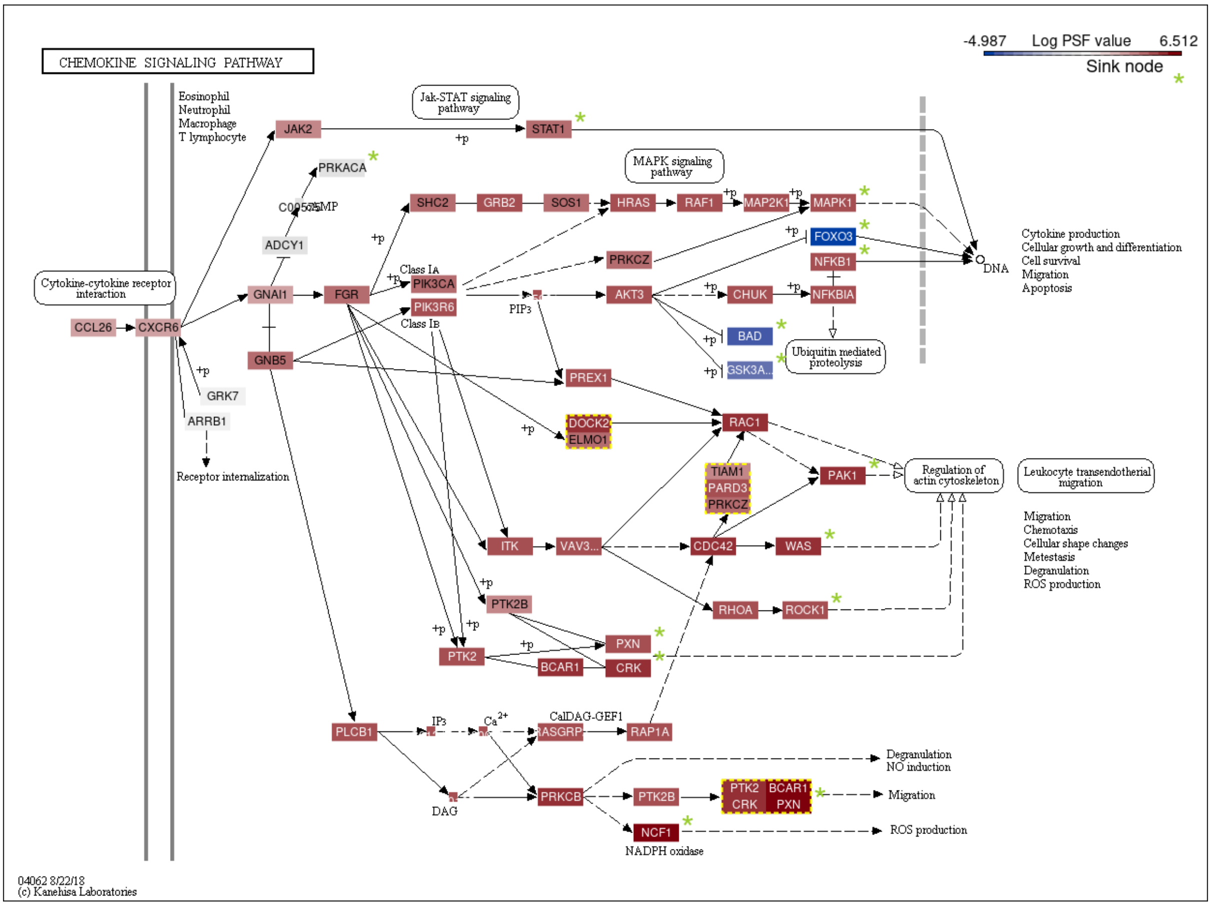

Figure S3. Chemokine signaling pathway color-coded with pathway activity values. The pathway is classified as activated with the SPIA algorithm, while there are three terminal nodes that are inhibited with the AKT3 gene.

A.

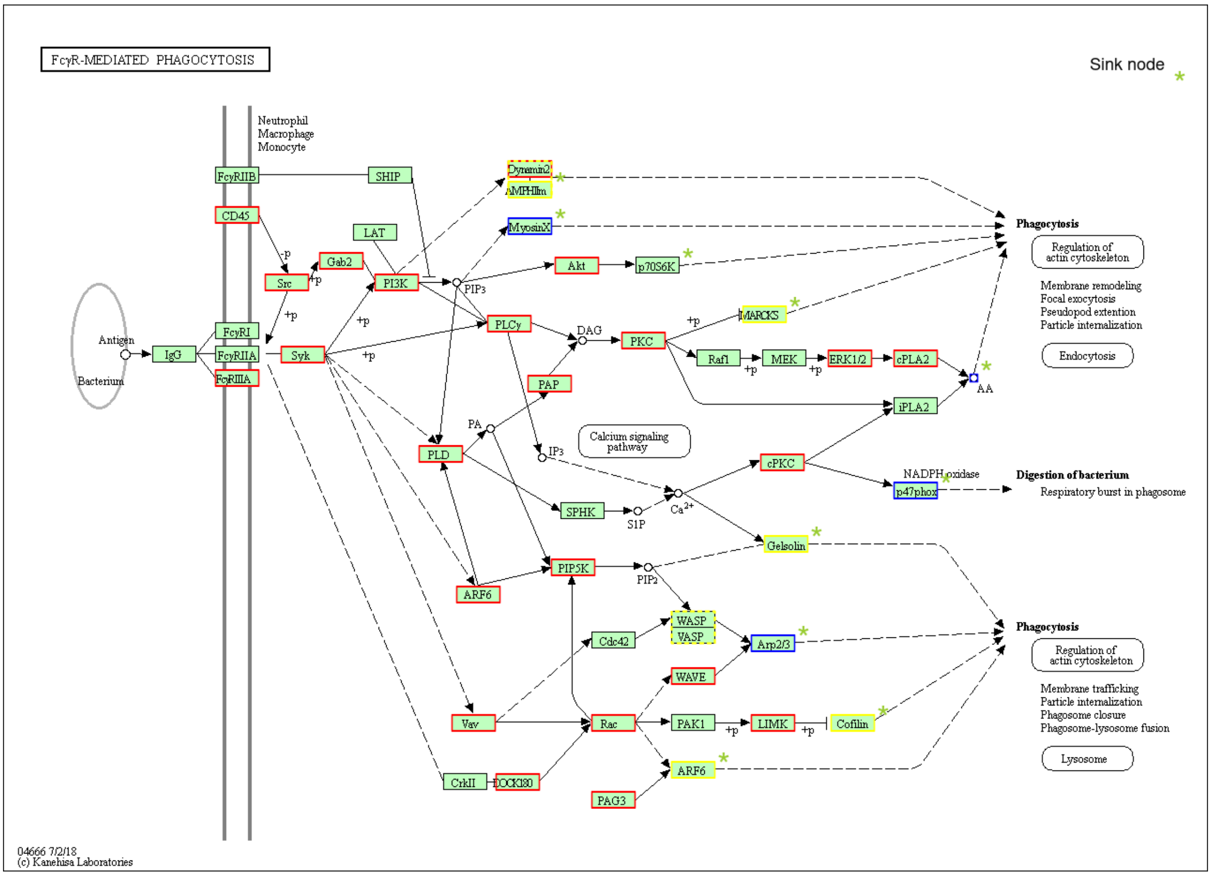

B.

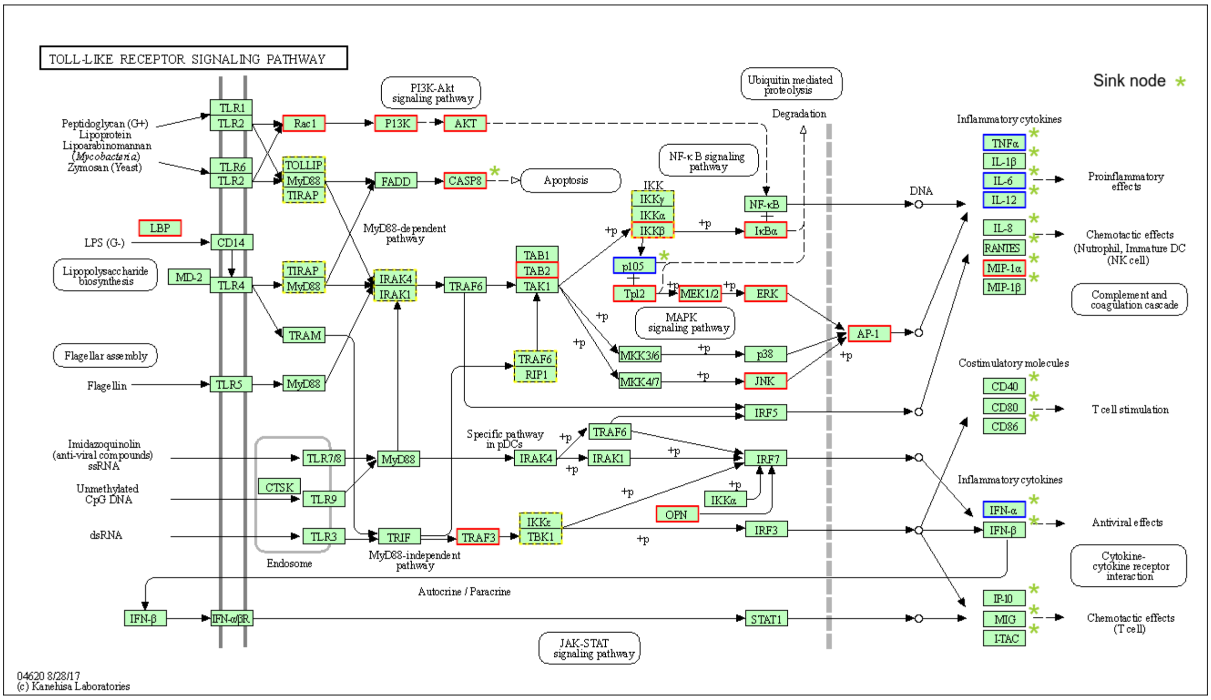

Figure S4. Two KEGG signaling pathways deregulated in lung cells. The first pathway is detected with both the PSF and ORA methods, while the second is only detected with the PSF algorithm. Differentially expressed genes/nodes are highlighted with red borders and deregulated terminal nodes with blue (yellow if the node is both differentially expressed and deregulated by the PSF algorithm). **A)** Fc gamma R mediated phagocytosis pathway,

detected with ORA where 33 genes are differentially expressed out of 91. The proportion of deregulated terminal nodes over the total is 0.9 (9 nodes out of 10). **B)** Toll-like receptor signaling pathway detected only with the PSF method where 16 genes were differentially expressed out of 104. The proportion of deregulated terminal nodes over the total is 0.27 (5 nodes out of 18). This demonstrates the sensitivity of the PSF algorithm to detect part of a deregulated pathway.

Supplementary table1. Top 5 cluster-associated features for each tissue type from gene and pathway activity clustering and top 5 enriched pathways based on gene level cluster specific significant genes.

Cluster/Tissue	Top genes	Top psf branches	Top enriched pathways
skeletalmuscle	MYF6	51-FoxO-signaling-pathway	Focal adhesion
skeletalmuscle	PPP1R27	56-FoxO-signaling-pathway	Arrhythmogenic right ventricular cardiomyopathy (ARVC)
skeletalmuscle	CACNG1	88-FoxO-signaling-pathway	Rap1 signaling pathway
skeletalmuscle	CHRNA1	27-PI3K-Akt-signaling-pathway	Endocytosis
skeletalmuscle	DDIT4L	50-FoxO-signaling-pathway	Regulation of actin cytoskeleton
breast	SLC7A10	8-ErbB-signaling-pathway	Focal adhesion
breast	LGALS12	7-JAK-STAT-signaling-pathway	Dilated cardiomyopathy (DCM)
breast	OXTR	52-Hedgehog-signaling-pathway	AMPK signaling pathway
breast	ANGPTL8	50-Hedgehog-signaling-pathway	Hypertrophic cardiomyopathy (HCM)
breast	ADIPOQ	24-ErbB-signaling-pathway	Cardiac muscle contraction
esophagusmucosa	CRNN	27-JAK-STAT-signaling-pathway	Rap1 signaling pathway
esophagusmucosa	SPRR3	29-B-cell-receptor-signaling-pathway	Focal adhesion
esophagusmucosa	SPRR2A	44-T-cell-receptor-signaling-pathway	Dilated cardiomyopathy (DCM)
esophagusmucosa	SPRR2D	60-cAMP-signaling-pathway	Axon guidance
esophagusmucosa	KRT6C	48-Rap1-signaling-pathway	Adherens junction
esophagusmuscularis	NKX6-1	27-p53-signaling-pathway	Hypertrophic cardiomyopathy (HCM)
esophagusmuscularis	IL6	110-cAMP-signaling-pathway	Arrhythmogenic right ventricular cardiomyopathy (ARVC)
esophagusmuscularis	SPP2	118-cAMP-signaling-pathway	Focal adhesion
esophagusmuscularis	FAM83D	61-Complement-and-coagulation-cascades	Dilated cardiomyopathy (DCM)
esophagusmuscularis	FOXF2	62-Complement-and-coagulation-cascades	PI3K-Akt signaling pathway
heart	NPPB	26-cAMP-signaling-pathway	Focal adhesion
heart	MYL7	7-cAMP-signaling-pathway	Cardiac muscle contraction
heart	TNNI3	8-Calcium-signaling-pathway	Alzheimer disease

heart	TBX20	5-cAMP-signaling-pathway	Axon guidance
heart	ACTC1	20-Calcium-signaling-pathway	Regulation of actin cytoskeleton
lung	SFTPA1	15-Hedgehog-signaling-pathway	Focal adhesion
lung	NKX2-1	26-Hedgehog-signaling-pathway	Dilated cardiomyopathy (DCM)
lung	SFTPA2	33-Hedgehog-signaling-pathway	PI3K-Akt signaling pathway
lung	SFTPC	114-cAMP-signaling-pathway	Phospholipase D signaling pathway
lung	NGB	15-AMPK-signaling-pathway	Hypertrophic cardiomyopathy (HCM)
prostate	PRAC1	69-cAMP-signaling-pathway	Focal adhesion
prostate	KLK4	60-AMPK-signaling-pathway	Protein processing in endoplasmic reticulum
prostate	HOXB13	31-Wnt-signaling-pathway	Axon guidance
prostate	KLK3	100-cAMP-signaling-pathway	Prostate cancer
prostate	KLK2	26-TGF-beta-signaling-pathway	Proteoglycans in cancer
skin	KRT2	47-ErbB-signaling-pathway	Dilated cardiomyopathy (DCM)
skin	KRT1	48-ErbB-signaling-pathway	Hypertrophic cardiomyopathy (HCM)
skin	CLEC2A	20-NOD-like-receptor-signaling-pathway	Arrhythmogenic right ventricular cardiomyopathy (ARVC)
skin	DCD	79-Ras-signaling-pathway	Fluid shear stress and atherosclerosis
skin	WFDC5	64-Complement-and-coagulation-cascades	GnRH signaling pathway
